# Sufficient conditions using liquid concentration profile to control formation and shape of lotus-type pores in solid

**DOI:** 10.1016/j.heliyon.2024.e26224

**Published:** 2024-02-14

**Authors:** Y.T. Ou, P.S. Wei

**Affiliations:** Department of Mechanical and Electro-Mechanical Engineering, National Sun Yat-Sen University, Kaohsiung, 80424, Taiwan

**Keywords:** Porosity, Pore shape, Solidification, Bubble entrapment, Lotus type pores, Isolated pores

## Abstract

Sufficient conditions to control solute transport across the cap responsible for the formation, development, and final shape of the lotus-type pores for different spatial variations of the partition coefficient, and the ratio between concentration in solid at the solidification front and concentration at a reference state near the top free surface during unidirectional solidification are presented in this study. Lotus-type porous material contemporarily used in micro-or nano-technologies strongly depend on distributions, orientations, and shapes of pores in solid. The model accounts for solute pressure in the pore affected by solute transport and balance of gas, capillary and hydrostatic pressures, and Sieverts' law or Henry's law at the bubble cap and top free surface. Solute transport across the cap accounts for rejection and convection-affected concentration at solidification front, and convection based on the reference state deviated from that at the top free surface. The resulting simultaneous systems of unsteady first-order ordinary differential equations are solved by MATLAB code. Changing rate of solute pressure in the pore responsible for entrapment and final length of lotus-type pores affected by volume expansion, and solute transport due to diffusion and rejection by the solidification front at the cap is also analyzed. The predicted shapes of lotus-type pores agree with algebraic expression confirmed by available experimental data.

## 1 Introduction

Traditional porous metal materials have different pore shapes and irregular arrangement, which often lead to stress concentration on the pore boundary and degradation of mechanical properties [1,2]. On the other hand, materials containing lotus-type pores as invented by Shapovalov [3] have attracted much attention, because they exhibit not only light-weight, air permeability, but also controllable anisotropy of thermal and mechanical properties. Lotus-type porous metal has higher stress plateau, tensile and compressive strength along the pore direction than perpendicular direction [[4], [5], [6], [7]]. Lotus-type porous materials are thus suitable for applications in heat sinks, filters, damping, energy and sound absorption, and biomedical devices. Lotus-type porous metals possess long cylindrical pores aligned in one direction fabricated by gasar process, which is a process of the ‘metal-gas eutectic transformation’, namely, the solidification of melt into a solid solution and a gas phase. Porosity and pore shape can be controlled by the partial pressures of dissolved and inert gases, solidification rate and alloying elements, etc. [8,9].

The pore length and radius, and inter-pore spacing generally decrease as pressured solute and inert gas and solidification rate increase. In order to increase porosity defined by Apprill et al. [10], Park et al. [11] fabricated lotus-type porous Al–Si alloys in the absence of the mushy zone, and found that the more the content of alloying elements, the higher the porosity of porous aluminum alloys. Ide et al. [12] had successfully fabricated lotus aluminum in a mixture of hydrogen and argon, as the solidification velocity decreased. Kim et al. [13] obtained lotus aluminum using thermal decomposition method in vacuum, and Liu et al. [14] decreased hydrogen pressure in Bridgman-type directional solidification method in vacuum.

Mechanisms of lotus-type pores primarily dominated by solute concentration fields can be numerically revealed by solving two-dimensional solute transport equations by Drenchev et al. [15], Yang et al. [16], and Iitsuka et al. [17]. More simple and clear understanding of the shape of lotus-type pores can be analytically revealed by steady-solute concentration field subject to a flat top bubble in liquid and Gibbs-Thomson equation in the gasar solidification as found by Liu et al. [18]. It was also found that there existed the maximum porosity versus partial hydrogen pressure together with inert gas, which was further confirmed by Liu et al. [14]. A more elaborate and complicated steady-state concentration field around a self-consistent shape of the bubble cap in gasar eutectic growth was also provided by Li et al. [19] using multiple scale expansion and matching method. Yoshimura et al. [20] conducted scale solute transport in radial direction within the concentration boundary layer ahead of the solidification front into pore. Diameter and inter-pore spacing were found to be inversely proportional product of solidification rate with square root of ambient pressure, agreeing with measurements. Wei and Lee [21] accounted for conservation of total solute content of a system including concentration boundary layers on the bubble and solidification front, and introducing mass transfer coefficient to remove detailed description of solute concentration field, necessary conditions for algebraic expressions of the final shapes of lotus-type pores or isolated pore were obtained.

In this study, the effects of spatial variations of partition coefficients at the solidification front, and ratio between concentration in solid at solidification front and concentration at the reference state near the top free surface on formation, development, and final shapes of lotus-type pores during unidirectional solidification are numerically predicted. Solute transfer across the bubble cap due to rejected solute by the solidification front dominates development of lotus-type pores. Previous work [21] provided necessary condition for lotus-type shape shapes by accounting for conservation of solute in the system at initial time and contact angle of 90°. Since a realistic process is accomplished by time-marching simulation, this study introduces appropriate sufficient conditions to find development and final shapes of lotus-type pores via solving simultaneous system of unsteady first-order ordinary differential equations governing pressure balance and physico-chemical equilibrium at the bubble cap and top free surface, and relevant solute transport from the concentration boundary layers on the cap and solidification front.

## 2 System model and governing equations

**Fig. 1:**
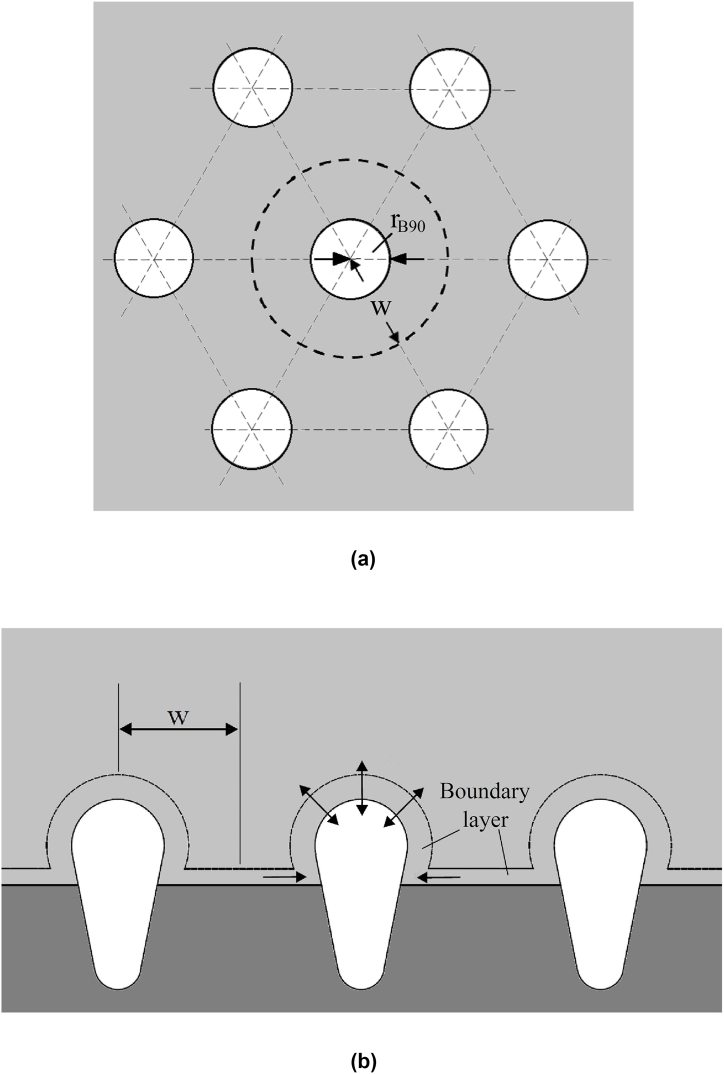
Schematic of (a) transverse and (b) longitudinal cross-section of lotus-type pores in this model.

Lotus-type pores entrapped by a solidification front are illustrated in Fig. 1 [21]. Concentration boundary layers exist on the solidification front and bubble cap. In view distinct solubility between solid and liquid, solute is rejected and accumulated ahead of the solidification front. Supersaturation results in morphological instability of the solidification front, nucleation, and sequential growth of bubbles. Inter-pore spacing thus can be scaled by wavelength of morphological instability [22]. Formation of lotus-type pores thus depends on solute transport across the bubble cap from concentration boundary layers on the cap and solidification front. Without loss of generality, the major assumptions are unidirectional and axisymmetric growth of pores initiated from spherical bubbles in static liquid on the solidification front. Force convection and free convection can be ignored [23,24]. Transient solute content in the pore can be simulated by

(1) $$\frac{d{\tilde{\rho }}_{g}{\tilde{V}}_{g}}{d\tilde{t}}={\tilde{A}}_{cr}{\tilde{h}}_{D}\left({\tilde{C}}_{\infty }-{\tilde{C}}_{c}\right)+\pi \eta \left({\tilde{w}}^{2}-{\tilde{R}}^{2}\;\sin^{2}\varphi_{B}\right)\tilde{U}\left({\tilde{C}}_{l}-{\tilde{C}}_{s}\right)$$

where terms on the right-hand side represent solute transport across the cap due to convection or diffusion in liquid, and solute rejected by the solidification front, respectively. Solute concentration in solid at the solidification front is related to that in liquid at the solidification front and at top free surface

(2) $${\tilde{C}}_{s}=k_{p}{\tilde{C}}_{l},{\tilde{C}}_{s}=\beta {\tilde{C}}_{\infty }$$

where parameter $k_{p}$ is partition coefficients at the solidification front, and β the ratio between concentration in solid at solidification front and concentration at a reference state near the free surface. Dimensionless solute concentration in liquid at the bubble cap and reference state are, respectively, determined by Sieverts' law imposed at cap and top free surface

(3) $$BoC_{c}=\frac{\sqrt{p_{g}}}{K_{Sc}},BoC_{\infty }=\frac{\sqrt{\gamma p_{a}}}{K_{S\infty }}$$

Substituting Eqs. (2), (3), dimensionless Eq. (1) becomes

(4) $$\frac{dp_{g}}{dt}=-\frac{p_{g}}{V}\frac{dV}{dt}+\frac{A_{cr}h_{D}}{V}\left(\frac{\sqrt{\gamma p_{a}}}{K_{S\infty }}-\frac{\sqrt{p_{g}}}{K_{Sc}}\right)+\frac{\pi \eta \left(w^{2}-R^{2}\;\sin^{2}\varphi_{B}\right)}{V}U\beta \frac{\sqrt{\gamma p_{a}}}{K_{S\infty }}\left(\frac{1}{k_{p}}-1\right)$$

Terms on the right-hand side of Eq. (4) thus represent changing rate of solute pressure in the pore affected by volume expansion or shrink, solute convection and solute rejected by the solidification front at the cap, respectively. Similarly, dimensionless solute concentrations at the cap and reference state subject to Henry's law at the cap and top free surface are, respectively

(5) $$BoC_{c}=\frac{p_{g}}{K_{Hc}},BoC_{\infty }=\frac{\gamma p_{a}}{K_{H\infty }}$$

Substituting Eq. (5) into dimensionless transient solute pressure in the pore from Eq (1) subject to Henry's law similarly gives

(6) $$\frac{dp_{g}}{dt}=-\frac{p_{g}}{V}\frac{dV}{dt}+\frac{A_{cr}h_{D}}{V}\left(\frac{\gamma p_{a}}{K_{H\infty }}-\frac{p_{g}}{K_{Hc}}\right)+\frac{\pi \eta \left(w^{2}-R^{2}\;\sin^{2}\varphi_{B}\right)}{V}U\beta \frac{\gamma p_{a}}{K_{H\infty }}\left(\frac{1}{k_{p}}-1\right)$$

Dependent variables in Eqs. (4), (6) can be solved by further including simultaneous first-order differential equations [25].

(7) $$\frac{ds}{dt}=\frac{\alpha_{s}}{\alpha_{l}}Le_{l}Ste_{s}\frac{\partial T_{s}}{\partial z}-\frac{\rho_{l}}{\rho_{s}}Le_{l}Ste_{l}\frac{\partial T_{l}}{\partial z}$$

(8) $$\frac{dR}{dt}=-2\left[\frac{dp_{g}}{dt}+Bo\left(-\frac{dh_{B}}{dt}+\frac{dz_{B}}{dt}\right)\right]{\left[p_{g}-p_{a}-Bo\left(h_{B}-z_{B}\right)\right]}^{-2}$$

(9) $$\frac{dy}{dt}=\frac{R}{y}\frac{dR}{dt}+\frac{ds}{dt}$$

(10) $$\frac{{dV}_{w}}{dt}=\pi r_{B}^{2}\frac{ds}{dt}$$

(11) $$\frac{dV}{dt}=\frac{dV_{w}}{dt}+2\pi R\left(R-y\right)\frac{dR}{dt}-\pi \left(R^{2}-y^{2}\right)\frac{dy}{dt}$$

(12) $$\frac{dh_{B}}{dt}=-\frac{ds}{dt}$$

(13) $$\frac{dz_{B}}{dt}=\frac{dR}{dt}-\frac{dy}{dt}$$

subject to initial conditions.

$p_{g0}$ = $Bo\left(h_{B0}-z_{B0}\right)+p_{a}+2$, *R = 1*, *s* = 0, $z_{B0}=1-\cos\;\varphi_{B0}$,

(14) $$y_{0}=\cos\;\varphi_{B0},V_{0}=\frac{4\pi }{3},V_{w0}=\frac{\pi }{3}{\left(1+\cos\;\varphi_{B0}\right)}^{2}\left(2-\cos\;\varphi_{B0}\right)$$

Eqs. (7), (8), (9), (10), (11), (12), (13) represent changing rates of solidification front location, apex radius based on Young-Laplace equation, Abel's equation of the first kind, pore volume below the solidification front, total pore volume, and liquid layer thickness and cap height. Given temperature gradients in liquid and solid at the solidification front, solidification rate can be algebraically obtained from Stefan boundary condition of Eq. (7) [26]. In view of decrease in solidification rate, mass transfer coefficient and partition coefficient are considered to decrease during solidification. Eqs. (4), (6), (7), (8), (9), (10), (11), (12), (13) with initial conditions of Eq. (14) were then solved by using MATLAB code with relative and absolute errors are, respectively, $10^{-7}$ and $1.3\times 10^{-6}$. Computed results at contact angle of 90° was sensitive to error bounds selected, which was thus checked and confirmed by analytical solutions [21], as can be seen later. Final length of the lotus-type pores can also be effectively analyzed by considering conservation of total solute content in the system composed of solute content in the pore and boundary layer on the bubble cap and solidification front

(15) $$\rho_{g}V+\int_{R}^{R+\delta_{R}}\left(C_{l}-C_{\infty }\right)2\pi r^{2}\left(1-\cos\;\varphi_{cr}\right)dr+\int_{s}^{s+\delta_{s}}\left(C_{l}-C_{\infty }\right)\pi \left(w^{2}-R^{2}\;\sin^{2}\varphi_{B}\right)dz=const\text{.}$$

Dimensionless length between locations at initial contact angle and contact angle of 90° gives

(16) $$l_{90}=\frac{1}{\left(\frac{2}{3}r_{B90}^{2}+\frac{1}{3}\sin^{2}\varphi_{B0}\right)p_{g90}}\{\frac{4p_{g0}}{3}-p_{g90}F_{3}\left(\varphi_{B0},r_{B90}\right)+\left(\frac{\sqrt{p_{g0}}}{K_{Sc}}-\frac{\sqrt{\gamma p_{a}}}{K_{S\infty }}\right)F_{1}\left(\varphi_{B0},h_{D0},U_{0}\right)-\left(\frac{\sqrt{p_{g90}}}{K_{Sc}}-\frac{\sqrt{\gamma p_{a}}}{K_{S\infty }}\right)F_{2}\left(r_{B90},h_{D90},U_{90}\right)+\frac{\sqrt{\gamma p_{a}}}{K_{S\infty }}F_{4}\left(\varphi_{B0},k_{p0},r_{B0},w,U_{0}\right)-\frac{\sqrt{\gamma p_{a}}}{K_{S\infty }}F_{5}\left(k_{p90},r_{B90},w,U_{90}\right)\}$$

where functions $F_{1}$, $F_{2}$, $F_{3}$, $F_{4}$, and $F_{5}$ are defined in Ref. [21].

## 3 Results and discussion

**Fig. 2:**
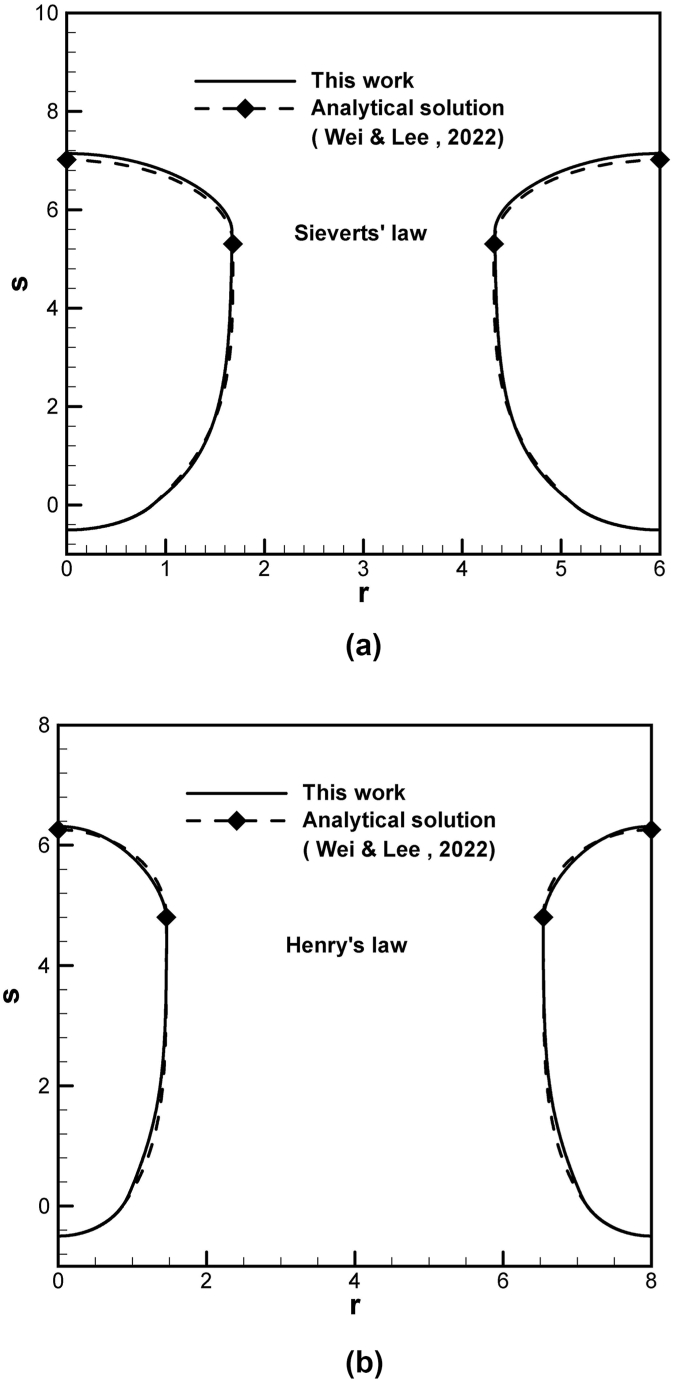
Predicted lotus-type pore shapes between analytical solution and this work subject to (a) Sieverts' law and (b) Henry's law.

The formation, development, and final shape of the lotus-type pores for different spatial variations of dimensionless partition coefficients and ratios between concentration in solid at the solidification front and concentration at a reference state near the top free surface during unidirectional solidification are provided and described on the universal phase diagrams [27]. Each pathline on phase diagrams is traced by solving simultaneous systems of unsteady differential Eqs. (4), (6), (7), (8), (9), (10), (11), (12), (13). Provided that factor γ is mole fraction, difference in solute concentrations at the bubble cap and top free surface satisfied by Sieverts' law from Eq. (3) and Young-Laplace equation gives $C_{c}-C_{\infty }∼\sqrt{p_{g}}-\left(K_{Sc}/K_{S\infty }\right)\sqrt{\gamma p_{a}}>0$. In accordance with the work [15,16], the factor can be generalized to $\gamma \equiv \gamma_{\infty }\gamma^{\prime }$, where $\gamma_{\infty }$ and $\gamma^{\prime }$ represent mole fraction of solute satisfied by Sieverts' law on the top free surface and concentration affected by convective or other sources, respectively. Factor γ therefore self-consistently allows solute concentration in liquid at the cap to be greater or less than that at the top free surface or the reference state, namely, $\gamma <1,C_{c}>C_{\infty }$ and $\gamma >1,C_{c}>C_{\infty }\;\text{or}\;C_{c}<C_{\infty }$. Moreover, solute concentration in liquid at the solidification front $C_{l}=\left(\beta /k_{p}\right)C_{\infty }$ from Eq. (2) can be relevantly affected by not only solute segregation at the solidification front but also convection at reference state [1]. Good comparison of pore shapes between analytical solution validated by available experimental data [21], and this work subject to Sieverts' law and Henry's law at the bubble cap is shown in Fig. 2(a) and (b), respectively. Slight difference between numerical result from this work and analytical solution [21] is attributed to interpolated approximation of the lotus-type pore shape between initial contact angle and contact angle at 90° [28].

**Fig. 3:**
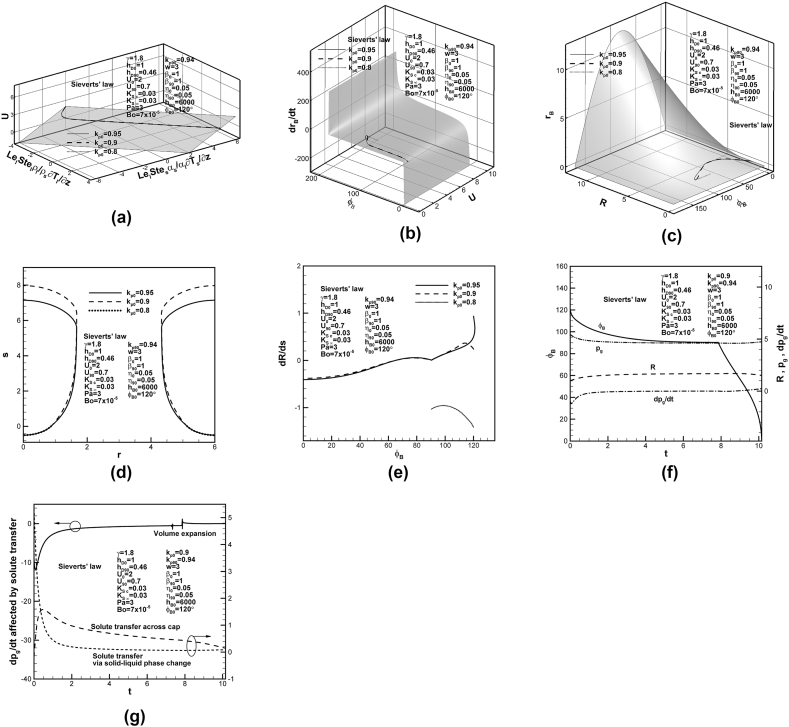
Predicted (a)–(c) lotus-type pore shape development on dimensionless universal phase diagrams, (d) final lotus-type pore shapes, and (e) bubble growth rate-to-solidification rate ratio versus contact angle for different partition coefficients at initial time, (f) dimensionless solute pressure and its changing rate, apex radius and contact angle versus time, and (g) dimensionless changing rate of solute pressure in pore affected by volume expansion, solute transport due to convection and rejection by solidification front on cap.

Dimensionless solidification rates for different partition coefficients at initial time in the case of Sieverts’ law imposed at the bubble cap and top free surface are shown on universal phase diagram in Fig. 3(a) under dimensionless coordinates of solidification rate, temperature gradients in solid and liquid at the solidification front. Together with dimensionless phase diagrams under coordinates of solidification rate, contact angle and growth rate of base radius, and apex and base radii, and contact angle of the cap, as shown in Fig. 3(b) and (c), respectively, lotus-type pores are traced and readily entrapped for higher dimensionless partition coefficients at initial time. The corresponding final shapes of lotus-type pores in solid are shown in Fig. 3(d). Length of entrapped lotus-type pores decreases as partition coefficient at initial time increases. This is because total solute content of the system decreases with solute content in the concentration boundary layer on the solidification front at initial time, governed by the last term on the left-hand side of Eq. (15) or the term involving $F_{4}$ related to reciprocal of partition coefficient at initial time of Eq. (16) [21]. Entrapment of lotus-type pores can also be confirmed by vanished bubble growth rate-to-solidification rate ratio at contact angle of 90° [29] for higher partition coefficients at initial time, as shown in Fig. 3(e). Interestingly, in contrast to monotonic decrease for partition coefficient at initial time of 0.95, bubble growth rate-to-solidification rate ratio enhances and then reduces its increasing rate in the very early stage for partition coefficient at initial time of 0.9. Variations of dimensionless solute pressure and its changing rates in the pore, contact angle and apex radius of the cap with time for partition coefficient at initial time of 0.9 are shown in Fig. 3(f). Referring to previous Fig. 3(e), decreasing rate of solute pressure in the pore rapidly enhances and then reduces to satisfy Young-Laplace equation in the very early stage. Apex radius and solute pressure approach constants at contact angle of 90°, leading to entrapment of lotus-type pores [29]. Vanished changing rate of solute pressure in the pore at contact angle of 90° can be controlled by volume expansion and solute transport across the cap, as shown in Fig. 3(g). Volume expansion evidently enhances and then reduces the decrease in solute pressure in the pore in the very early and late stages, respectively. Since difference in solute concentrations in liquid at the cap and reference state $\sqrt{p_{g0}}-\sqrt{\gamma p_{a}}\approx \sqrt{3+7\times 10^{-5}\times 6\times 10^{3}+2}-\sqrt{1.8\times 3}>0$, outward solute diffusion from the pore to surrounding liquid further enhances the decrease in solute pressure in the pore in the very early stage. As time increase, solute convection or diffusion in liquid changes direction from surrounding liquid into pore, enhancing and then reducing its effect on increase of solute pressure in the pore. Solute transport across the cap due to rejected solute by the solidification front monotonically reduces its effect on increase in solute pressure in the pore. As changing rate of solute pressure in the pore affected by volume expansion and inward solute diffusion in liquid and rejected solute by the solidification front vanishes, complete entrapment starts to occur at contact angle of 90° around dimensionless time of 8 (see Fig. 3(f)).

**Fig. 4:**
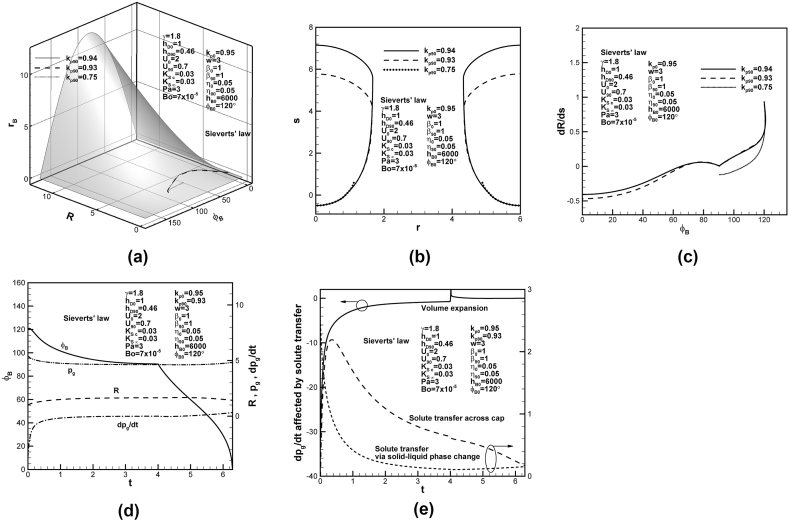
Predicted (a) lotus-type pore shape development on dimensionless universal phase diagram, and (b) final lotus-type pore shapes, and (c) bubble growth rate-solidification rate ratio versus contact angle for different partition coefficients at contact angle of 90°, (d) dimensionless solute pressure and its changing rate, apex radius, and contact angle versus time, and (e) dimensionless changing rate of solute pressure in pore affected by volume expansion, solute transport due to convection and rejection by solidification front on cap.

Dimensionless phase diagrams of Fig. 4(a) shows the development and entrapment of lotus-type pores in solid for different partition coefficients at contact angle of 90°. Lotus-type pores can be formed for higher dimensionless partition coefficients at contact angle of 90°. The corresponding final shapes of lotus-type pores are shown in Fig. 4(b). Length of entrapped lotus-type pores increases with partition coefficient at contact angle of 90°. As can be revealed from the last term on the left-hand side of Eq. (15) or term involving $F_{5}$ related to reciprocal of partition coefficient at contact angle of 90° in Eq. (16), increase in partition coefficient at contact angle of 90° decreases solute content in the concentration boundary layer on the solidification front at contact angle of 90°. To maintain total solute content of the system, solute content in the pore increases, resulting in final length of lotus-type pores to increase with partition coefficient at contact angle of 90°. Fig. 4(c) shows that bubble growth rate-to-solidification rate ratio for higher partition coefficients at contact angle of 90° monotonically decrease in the early stage. Lotus-type pores are thus readily formed for higher partition coefficient at contact angle of 90°. Time-dependent dimensionless solute pressure and its changing rate in the pore, contact angle and apex radius of the cap are shown in Fig. 4(d). Dimensionless apex radius and solute pressure in the pore rapidly increases and decreases in the early stage, respectively. Lotus-type pores are completely entrapped as solute pressure in the pore and apex radius approach constants at contact angle of 90°. Fig. 4(e) shows that volume expansion reduces the decrease in dimensionless solute pressure in the pore during solidification. Outward solute transfer on the bubble cap due to solute diffusion in liquid, as discussed previously, further enhances the decrease in solute pressure in the pore in the very early stage. Inward solute diffusion then enhances and then reduces the increase of dimensionless solute pressure in the pore in early and late stage, respectively. Solute transport across the cap due to rejected solute by the solidification front gradually reduces increase in dimensionless solute pressure in the pore. As changing rate of dimensionless solute pressure in the pore affected by volume expansion and inward solute transport across the cap from liquid diffusion and solute rejected by the solidification front vanishes, lotus-type pores start to be completely entrapped at dimensionless time of around 4.

**Fig. 5:**
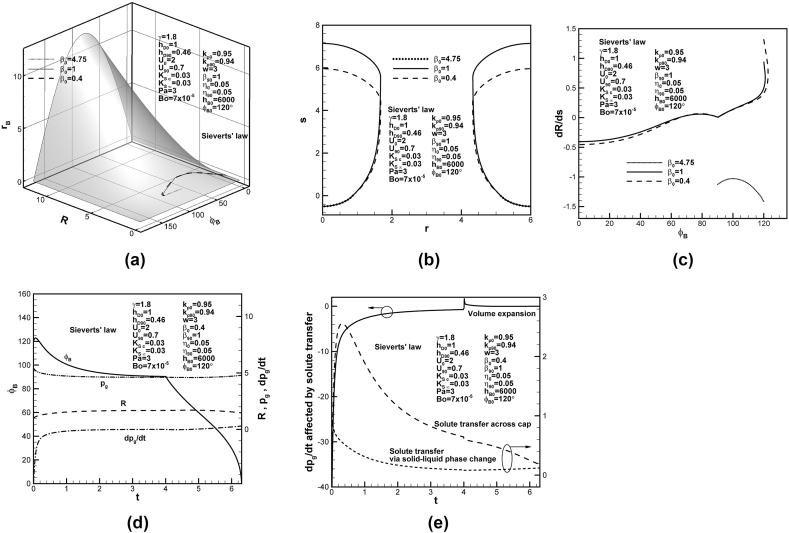
Predicted (a) lotus-type pore shape development on dimensionless universal phase diagram, (b) final lotus-type pore shapes, and (c) bubble growth rate-solidification rate ratio versus contact angle for different ratios between solid concentration at solidification front and liquid concentration at reference state at initial time, (d) dimensionless solute pressure and its changing rate, apex radius, and contact angle versus time, and (e) dimensionless changing rate of solute pressure in pore affected by volume expansion, solute transport due to convection and rejection by solidification front on cap.

The effects of the ratio between concentration in solid at solidification front and concentration at the reference state at initial contact angle on the development and final shape of lotus-type pores are shown in Fig. 5(a) and (b), respectively. The decrease in the ratio between concentration in solid at solidification front and concentration at reference state enhances the entrapment while reduces length of the lotus-type pores. The decrease in the lotus-type pore length is attributed to that total solute content of the system decreases with solute content in the concentration boundary layer on the solidification front at initial time (see Eq. (4)). Evidently, bubble growth rate-to-solidification rate ratio vanishes at contact angle of 90° for lower ratio between concentration in solid at solidification front and concentration at reference state at initial time, as shown in Fig. 5(c). Similar to previous Fig. 4(d) and (e), the variations of dimensionless solute pressure and its changing rate in the pore, contact angle and apex radius of the cap with time, and changing rates of dimensionless solute pressure in the pore affected by volume expansion, solute diffusion in liquid and rejected solute by the solidification front are shown in Fig. 5(d) and (e), respectively.

**Fig. 6:**
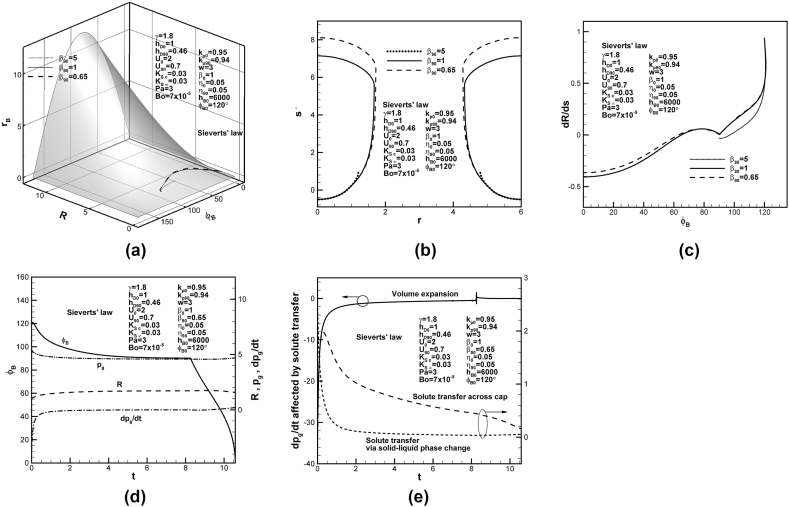
Predicted (a) lotus-type pore shape development on dimensionless universal phase diagrams, (b) final lotus-type pore shapes, and (c) bubble growth rate-solidification rate ratio versus contact angle for different ratios between solid concentration at solidification front and liquid concentration at reference state at contact angle of 90°, (d) dimensionless solute pressure and its changing rate, apex radius, and contact angle versus time, and (e) dimensionless changing rate of solute pressure affected by volume expansion, solute transport due to convection and rejection by solidification front on cap.

Decrease in the ratio between concentration in solid at solidification front and concentration at reference state at contact angle of 90° enhances entrapment, while increases length of lotus-type pores, as shown in Fig. 6(a) and (b), respectively. The ratio between concentration in solid at solidification front and concentration at reference state at contact angle of 90° decreases with solute content in the concentration boundary layer on the solidification front at contact angle of 90°. To maintain total solute content in the system, solute content in the pore or final length of lotus-type pores increases, as shown in Fig. 6(b). Bubble growth rate-to-solidification rate ratios at contact angle of 90° vanish for lower ratios between concentration in solid at the solidification front and concentration at reference state, as shown in Fig. 6(c). Time-dependent dimensionless solute pressure and its changing rate in the pore, contact angle and apex radius of the cap with time, and changing rates of dimensionless solute pressure in the pore affected by volume expansion, solute diffusion in liquid and rejected solute by the solidification front are similar to previous Fig. 5(d) and (e), and shown in Fig. 6(d) and (e), respectively.

**Fig. 7:**
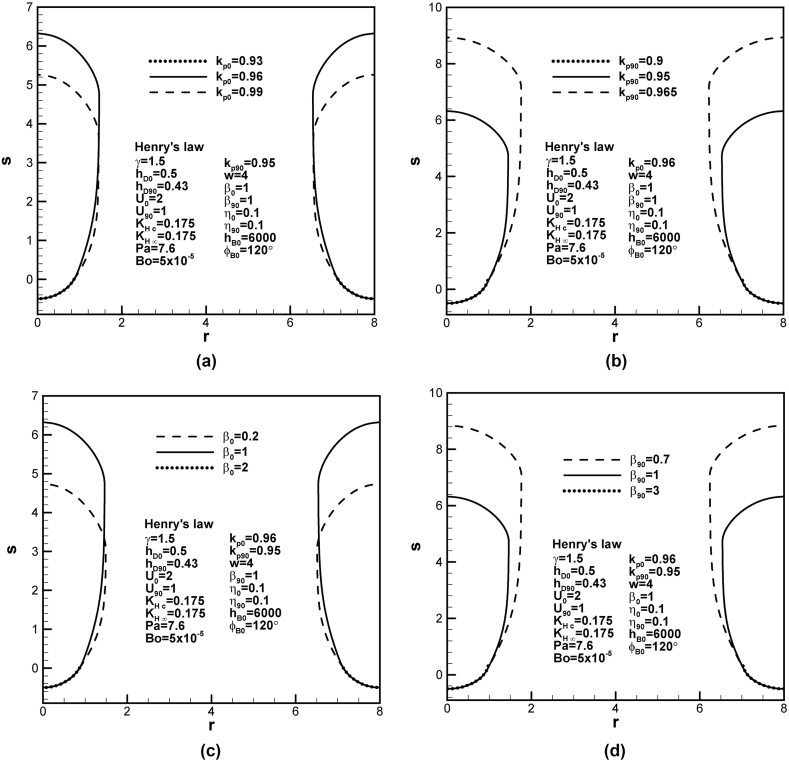
Final lotus-type pore shapes for different (a) and (b) partition coefficients at initial time and contact angle of 90°, and (c) and (d) ratios between solid concentration at solidification front and liquid concentration at reference state at initial time and contact angle of 90° subject to Henry's law at cap.

Similar to Sieverts' law, the effects of partition coefficients and ratios between concentration in solid at the solidification front and concentration at reference state at initial time and contact angle of 90° on final shapes of lotus-type pores subject to Henry's law constant are shown in Fig. 7(a)–(d).

## 4 Conclusions

The conclusions drawn are as follows:

1. Sufficient conditions to control the development and shape of lotus-type pores can be obtained via solving simultaneous systems of unsteady first-order ordinary differential equations governing solute pressure in the pore, Stefan condition, Abel's equation, Young-Laplace equation and physico-chemical interfacial equilibrium, pore volume, liquid layer thickness and bubble shape. Solute transport across the cap responsible for development of lotus-type pores relevantly accounts for rejection and convection-affected concentration on the solidification front, and convection based on a reference state rather than the top free surface.
2. A realistic process is accomplished by time-marching simulation via introduction of sufficient conditions, rather than necessary conditions considering conservation of solute in the system at initial time and contact angle of 90°.
3. Increasing rate and decreasing rate of dimensionless solute pressure in the pore due to volume expansion rapidly reduces and then enhances, respectively, in the very early stage for higher partition coefficient at initial time of 0.9. This is because increasing rate of apex radius rapidly increases and then decreases in the very early stage. Decreasing rate of solute pressure in the pore, however, monotonically decreases due to monotonic decrease in increasing rate of apex radius for partition coefficient at initial contact angle of 0.95. Solute concentration in liquid at the cap to be greater than that at the reference state results in outward solute diffusion to further enhance the decrease in solute pressure in the pore in the very early stage. Decrease in solute pressure in the pore is then reduced by inward solute transport due to convection and rejection by the solidification front. Lotus-type pores starts to form as dimensionless solute pressure in the pore vanishes at contact angle of 90°.
4. Decreasing rate of solute pressure in the pore affected by volume expansion rapidly reduces for higher partition coefficient at contact angle of 90° in the early stage. This is attributed to rapid decrease in the increase of apex radius. Outward solute diffusion further decreases dimensionless solute pressure in the pore in the very early stage. Dimensionless solute pressure in the pore then reduces its decrease rate and enhances increasing rate as solute diffusion becomes inward in early and late stage, respectively. Lotus-type pores are completely entrapped as changing rate of solute pressure in the pore due to volume expansion, inward solute transport from rejected solute by the solidification front and diffusion in liquid at the cap vanishes at contact angle of 90°.
5. The variations of dimensionless solute pressure in the pore affected by volume expansion, solute transfer due to diffusion in liquid and rejection by the solidification front at the cap with time subject to different ratios between concentration in solid at solidification front and concentration at the free surface at initial time and contact angle of 90° are similar to those of partition coefficient at contact angle of 90°.
6. Lotus-type pores readily form as partition coefficients at initial contact angle and contact angle of 90° increase, whereas ratios between concentration in solid at solidification front and concentration at reference state at initial time and contact angle of 90° decrease.
7. Final length of lotus-type pores increases as partition coefficient at initial contact angle decreases. This is because of increase in total solute content in the system due to enhanced solute content in the concentration boundary layer on the solidification front at initial time. A decrease in partition coefficient at contact angle of 90° increases solute content in the concentration boundary layer on the solidification front at contact angle of 90°. To maintain total solute content in the system, solute content in the pore decreases with length of the lotus-type pores at contact angle of 90°.
8. Final length of lotus-type pores increases as the ratio of concentration in solid at the solidification front and concentration at reference state at initial contact angle increases, whereas that at contact angle of 90° decreases. Total solute content of the system increases with solute content of concentration boundary layer on the solidification front as ratio of concentration in solid at the solidification front and concentration at top surface at initial time increases. To maintain total solute content in the system, decrease in solute content in the concentration boundary layer on the solidification front with the ratio of concentration in solid at the solidification front and concentration at top free surface increases solute content and length of the pore at contact angle of 90°.
9. The effects of Sieverts' law on formation and shape of lotus-type pores are similar to those of Henry's law.

## Data availability

No data was used for the research described in the article.

## Funding

This work was supported by 10.13039/501100004663MOST 103-2221-E-110-035-MY3, ROC.

## Declaration of competing interest

The authors declare that they have no known competing financial interests or personal relationships that could have appeared to influence the work reported in this paper.

## Nomenclature

$A_{cr}$: surface area $A_{cr}={\tilde{A}}_{cr}/{\tilde{R}}_{0}^{2}$
*Bo*: Bond number, $Bo\equiv {\tilde{\rho }}_{l}g{\tilde{R}}_{0}^{2}/\sigma$
C: solute concentration, $C=\tilde{C}{\tilde{R}}_{u}{\tilde{T}}_{m}/{\tilde{\rho }}_{l}g{\tilde{R}}_{0}$
*D*: solute diffusivity
$F_{1}$, .., $F_{5}$: coefficient functions
*g*: gravity acceleration
$h_{B}$: liquid depth
$h_{D}$: mass transfer coefficient, $h_{D}={\tilde{h}}_{D}{\tilde{R}}_{0}/{\tilde{D}}_{l}$
$K_{H}$: Henry's law constant, $K_{H}={\tilde{K}}_{H}/{\tilde{R}}_{u}{\tilde{T}}_{m}$
$k_{p}$: partition coefficient
$K_{S}$: Sievert's law constant, $K_{S}={\tilde{K}}_{S}\sqrt{\sigma /{\tilde{R}}_{0}}/{\tilde{R}}_{u}{\tilde{T}}_{m}$
l: pore length
*L*: latent heat
*Le*: Lewis number, $Le\equiv \tilde{\alpha }/\tilde{D}$
*p*: pressure, $p\equiv \tilde{p}{\tilde{R}}_{0}/\sigma$
*r*: coordinate, $r\equiv \tilde{r}/{\tilde{R}}_{0}$
*R*: apex radius, $R\equiv \tilde{R}/{\tilde{R}}_{0}$
$R_{u}$: universal gas constant
*s*: solidification front location $s\equiv \tilde{s}/{\tilde{R}}_{0}$
*Ste*: Stefan number, $Ste\equiv {\tilde{c}}_{p}{\tilde{T}}_{m}/\tilde{L}$
*t*: time, $t=\tilde{t}\tilde{D}/{\tilde{R}}_{0}^{2}$
*T*: temperature, $T\equiv \tilde{T}/{\tilde{T}}_{m}$
*U*: solidification rate $U\equiv \tilde{U}{\tilde{R}}_{0}/{\tilde{D}}_{l}$, *U = ds/dt*
*V*: volume, $V=\tilde{V}/{\tilde{R}}_{0}^{3}$
*w*: Inter-pore spacing
*y*: $y\equiv \tilde{R}\cos\;\varphi_{B}/{\tilde{R}}_{0}$
*z*: coordinate, $z\equiv \tilde{z}/{\tilde{R}}_{0}$

**Greek letter**
α: thermal diffusivity
β: ratio of solid concentration at solidification front and liquid concentration at reference state
$\delta_{R},\delta_{S}$: Concentration boundary layer thickness on bubble cap and solidification front
γ: factor accounting for concentration at reference state in liquid deviated from that at top free surface
η: solute transport parameter
σ: surface tension
ρ: density
φ: inclination or contact angle

**Subscripts**
*a*: ambient
*B*: base
*c*: cap
*cr*: critical location between boundary layers
*g*: gas
l: liquid
*m*: melting temperature
*s*: solid
*w*: pore volume below solidification front
*0*: initial state
*90*: contact angle of 90°
∞: far from solidification front

**Superscripts**
∼: dimensional quantity
